# Detection of Auto-Immune Disease using Deep Learning Techniques

**DOI:** 10.31138/mjr.060624.doa

**Published:** 2025-03-31

**Authors:** B Subramanya, Divya B Shivanna, Nithin Raj G, Pratham S Prabhu, Mohammed Yaseer, Roopa S Rao

**Affiliations:** 1 Department of Computer Science and Engineering, Faculty of Engineering & Technology, Ramaiah University of Applied Sciences, Bengaluru, India; 2 Department of Oral Pathology, Faculty of Dental Sciences, Ramaiah University of Applied Sciences, Bengaluru, India

**Keywords:** autoimmune disorders, HEp-2 cells, Detectron2 model, YOLOv8n model, instance segmentation

## Abstract

**Objective:**

The diagnosis of autoimmune disorders, particularly through the Anti-Nuclear Antibodies (ANA) Indirect Immunofluorescence (IIF) test utilising human epithelial type-2 (HEp-2) cells, presents a formidable challenge due to the subjective nature of pathologists’ analysis. In response, this study proposes an innovative automated approach that integrates deep learning, advanced image processing, guided Hep-2 Cell, and mitotic cell instance segmentation.

**Methods:**

Leveraging the ICPR 2016 dataset for training and evaluation, this research encountered an initial challenge of dataset imbalance, with a significantly lower number of mitotic cells compared to HEp-2 Homogenous cells. To overcome this, data augmentation techniques were strategically employed to ensure a balanced representation.

**Results:**

In Experiment 1, the Detectron2 model achieved an overall mean Average Precision of 54% for segmentation masks and 55% for bounding boxes. In Experiment 2, the YOLOv8n model demonstrated an impressive overall Mean Average Precision score of 94% for bounding boxes and 93% for segmentation masks, showcasing its exceptional efficacy in detecting HEp-2 cells and mitotic cells. The instance segmentation provided a more granular analysis, revealing the count of cells in each class, further highlighting the model’s proficiency in diagnosing autoimmune diseases.

**Conclusion:**

This study establishes a reliable and automated method for HEp-2 Homogenous cell detection, addressing the ongoing challenges in autoimmune disease diagnosis and contributing significantly to the ongoing revolution in this critical field.

## INTRODUCTION

In the rapidly evolving field of autoimmune disease diagnostics, this research paper presents an automated approach for Human Epithelial-type 2 cell segmentation. It draws inspiration and information from important recent works. Because autoimmune illnesses are complex, especially when combined with the indirect immunofluorescence ANA test,^1^ utilising state-of-the-art techniques. are required to improve accuracy and efficiency in diagnosis. The project expands on the groundwork established by Hai Xie and colleagues, who developed an adversarial network-based segmentation-guided HEp-2 cell classification method.^2^

To broaden the scope, exploring deep learning in HEp-2 cell analysis entails putting up an automated segmentation and classification system. Their research, which comes from Changsha Central Hospital, highlights how deep learning can revolutionise the accuracy of antinu-clear antibody picture analysis.^3^

The system’s goal is to close the gap between image processing and life sciences by offering a thorough automated analysis that complies with industry standards for the automatic segmentation and classification of anti-nuclear antibody images using deep learning techniques to jointly classify intensities and segment specimens on HEp-2 images.^4^ Their research from the University of Salerno adds to the field of HEp-2 cell analysis’s ongoing evolution.^5–12^ Additionally, the exploration of image segmentation for human epithelial type 2 cells,^12^ concentrates on modified quantum entropy-based indirect immune fluorescence, adding a special element to the variety of segmentation techniques used for HEp-2 cell images.^13^

## MATERIALS AND METHOD

### Dataset

**Figure 1.**
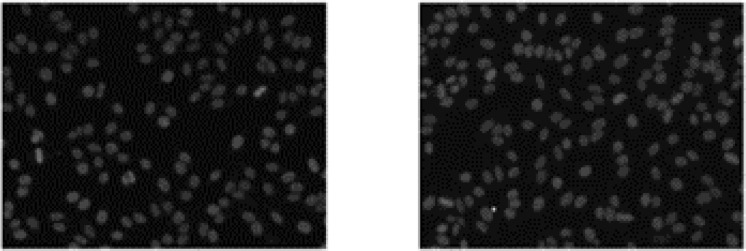
Greyscale images of autoimmune disease (Hep2 cells).

The HEp-2 Cells Classification contest dataset was used to assess the proposed algorithm. The International Conference on Pattern Recognition 2016 (ICPR 2016) organised the contest. This dataset comprises a total of 104 images, specifically categorised into two classes: homogenous cells and mitotic cells, as shown in **Figure 1**. To ensure a robust evaluation, the dataset was systematically partitioned into three distinct sets: a training set accounting for 69%, a validation set for 21%, and a testing set for 10% of the total dataset. The training set played a pivotal role in the iterative refinement of the network by adjusting weights, whereas the validation set served the critical purpose of preventing overfitting during the training process. Finally, the testing set served as a novel data source for the network, offering an independent assessment of its detection capabilities when faced with previously unseen data. This partitioned evaluation strategy enhances the algorithm’s generalisability and underscores its efficacy in handling diverse datasets.

### Algorithm

Following the initial stage of feature extraction in the deep learning models during experiments 1 and 2, numerous bounding boxes are generated as predictions.^14^ These models then assess the presence of objects within these predicted bounding boxes using a threshold known as the Intersection over Union (IoU) threshold. When the Intersection over Union (IoU) value of a predicted bounding box surpasses the predetermined threshold established in experiments 1 and 2, a more stringent bounding box is employed to accurately delineate the object. This process culminates in the creation of a final bounding box.

**Figure 2.**
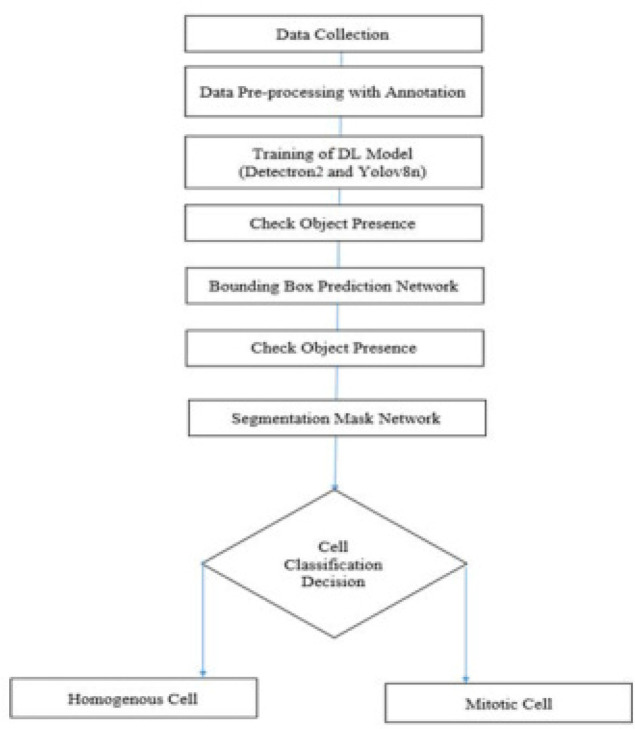
Algorithm.

Subsequently, pixel-wise segmentation masks are predicted for the identified objects. These objects are further classified into distinct categories, specifically, homogenous cells or mitotic cells, as shown in **Figure 2**. This comprehensive approach ensures precise object localisation and classification within the given context.^15,16^

### Pre-Processing

**Figure 3.**
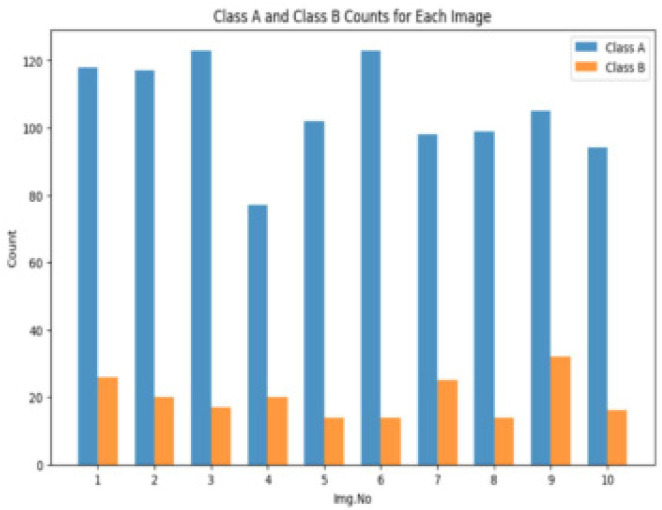
**(left).** Class Imbalance between Class A and Class B.

During the preprocessing stage of the study, after selection of a dataset of scanned images of human epithelial cells, added thoughtful annotations to make the deep-learning models easier to train. The priority was given to the standardisation of the images to a consistent resolution because the dataset was highly varied, as shown in **Figure 3**. The normalisation was used to ensure that the pixel values were within a consistent range and that the YOLOv8 would remain robust. The model generalisation was also improved. To generate segmentation masks, which were essential for training the models to recognise patterns associated with diseases, the annotations were processed and used. After that, the dataset was divided into testing, validation, and training sets, paying close attention to correcting any class imbalances. For categorical disease labels, one-hot encoding was used, and the pre-processed data was saved virtually for later training. Focusing on precision and effectiveness in the identification of autoimmune disorders, the computerised approach for the division and categorisation of human epithelial type-2 cells, made possible by this extensive preprocessing pipeline, which was inspired by and built upon the methodology of previous studies.^14,17^

To provide a clear understanding of the class imbalance, a bar chart was presented comparing the counts of Class A and Class B for a subset of ten randomly selected images from the dataset. The decision to analyse a subset of ten images instead of the entire data-set was made to streamline the presentation of results. A comprehensive analysis of the entire dataset would be impractical within the scope of this paper, and the chosen subset provides a representative snapshot for discussion.

The chart visually demonstrates the potential class imbalance, highlighting the necessity of addressing this issue through augmentation techniques during model training.

To address the challenge of class imbalance within the dataset, data augmentation techniques were employed, specifically focusing on random rotation and flipping to artificially increase the diversity of the training set.

The decision to incorporate random rotation and flipping as augmentation methods is based on their demonstrated effectiveness in improving the model’s resilience and capacity for generalisation. Random rotation exposes the model to cells in different orientations, introducing an important kind of invariance, especially in real-world situations where there can be large variations in the orientation of cells in microscopic images. The goal is to provide the model with the capacity to identify cells regardless of their orientation in space by training it with rotated examples, thereby fostering a more robust output. The aim to replicate various imaging conditions is the driving force behind the addition of flipping in addition to rotation. In real-world applications, mirrored or flipped representations could result from changes in lighting, sample preparation, or imaging technology. By allowing the model to learn and adjust to these variations through the addition of flipped instances to the dataset, its ability to identify cells in various imaging scenarios is improved, leading to better performance in real-world applications by ensuring that it generalises well when faced with new data.

It is crucial to acknowledge the potential limitations associated with extreme augmentations. In the current study, extreme augmentations were intentionally avoided to mitigate these potential limitations. The chosen augmentation strategies, namely random rotation and flipping, were applied judiciously to strike a balance between diversifying the training dataset and ensuring that the synthetic variations introduced closely represent the expected variations in real-world scenarios. By not employing extreme augmentations, the aim was to maintain the relevance and authenticity of the augmented data, thereby avoiding bias.

### Image Annotation

**Figure 4.**
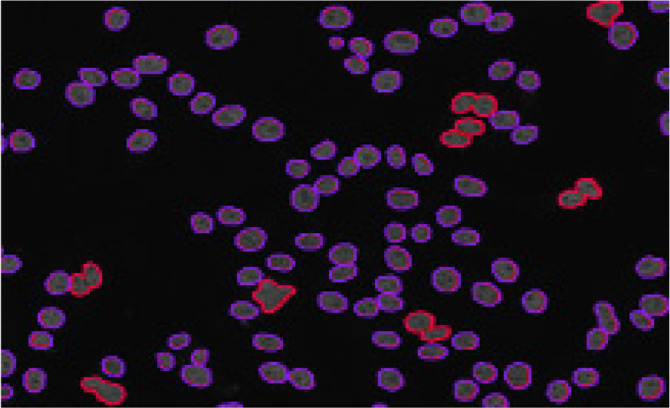
**(above).** Annotated image. The image shows the annotation of images into two types: affected homogeneous-rim cells (purple) and affected homogeneous rim-mitotic cells (red).

Image annotation is the procedure of marking images to facilitate the training of machine learning and deep learning models.^18,19^ It requires human annotators to employ an image annotation tool for labelling images or adding pertinent information, such as attributing appropriate classes to distinct entities within an image, as shown in **Figure 4**. The study commenced with the judicious annotation of HEp2 cell images (dataset name is required), a critical step achieved by utilisation of the Roboflow tool, categorising cells into two distinct classes: affected homogeneous rim cells and affected homogeneous rim-mitotic cells. The annotation process involved capturing disease-relevant characteristics to differentiate between these classes.

For affected homogeneous rim cells, those unaffected by the mitosis process, annotations emphasised the distinctive features indicative of a single affected cell. Conversely, affected homogeneous rim-mitotic cells, engaged in the intricate dance of cellular division, were annotated to highlight the observable mitosis process, resulting in the visual semblance of two connected cells.

The output from the annotation process serves as a cornerstone of the research. The predictions generated offer a wealth of information, encompassing coordinates (x, y) and dimensions (width, height) that precisely delineate the annotated regions. This detailed spatial data is complemented by a confidence score, indicating the model’s certainty in its predictions. The “class” attribute further categorises the cells, offering insights into their nature. Specifically, it distinguishes whether a cell is an affected homogeneous rim cell undergoing a rim-mitosis process.

In the realm of computer vision,^19,20^ object detection stands as a formidable tool, capable of not only identifying objects in an image but also providing precise coordinates and confidence levels for each prediction.^20^ When applied to the field of medicine, particularly in studying autoimmune diseases, its significance is amplified. Object detection aids in automatically locating and understanding objects in medical images, such as autoimmune disease cells and mitotic cells, offering a clearer insight into the internal workings of the body and significantly improving diagnostic capabilities .^21,22^

Image segmentation, another robust technique in computer vision, goes beyond object detection by breaking down entire images into meaningful sections or regions.^15^ This detailed mapping is particularly beneficial in medical imaging, highlighting and defining specific structures like organs or cells.^23,24^ In the context of autoimmune disease cell detection and segmentation, becomes a valuable tool, offering a comprehensive view of cellular structures and enabling more precise analysis and diagnosis.

Within the realm of image segmentation, semantic segmentation classifies each pixel in an image, assigning labels based on the represented objects.^24^ On the other hand, instance segmentation takes precision to the next level by outlining individual objects down to the pixel, creating detailed segmentation masks.^24^ This fine-grained approach, especially in the case of spotting autoimmune disease cells, proves crucial for accurate diagnostics.

The YOLOv8n (You Only Look Once version 8 with improvements) technology emerges as a cutting-edge solution in the world of computer vision, specifically tailored for segmentation tasks. Acting as an efficient detective, YOLOv8n excels in spotting and outlining individual objects in an image, predicting pixel-level boundaries, and creating segmentation masks. Thanks to the distribution focal loss feature, its adaptability to imbalanced datasets ensures robust performance in handling diverse cell types in medical images.

### Deep Learning Model for Detection of Hep2 Cells

The detection of autoimmune disease cells (HEp2 cells) images is achieved through a robust deep-learning algorithm of YOLOv8n and Detectron2.^5,6^ These models were carefully chosen for their exceptional ability to detect multiple objects, such as HEp2 cells, with great accuracy and speed.^6^ Additionally, they possess the capability to determine the confidence level of each detected bounding box using the final neural network layer.^6^ Not only that, but they also have the unique capability to detect objects of varying sizes, thanks to their trained bounding box coordinates regression function. And let’s not overlook YOLOv8n, renowned for its incredibly fast inference speed, which makes it a good choice for real-time applications where speed is essential.

### Architecture of YOLOv8n

YOLOv8n employs a unified neural network architecture with two stages. In the initial stage, a CNN-based backbone network makes up the first stage, which is designed to extract deep distinctive features extracted from the input image at a higher level of abstraction. In the second stage, the bounding box coordinates surrounding the HEp2 cells are detected by the head predictor. In the past few years, an additional stratum called the neck layer has been introduced, situated specifically amid the head network and the backbone. The neck network features numerous deep learning routes, encompassing both upward and downward directions. Various convolutional layers within deep learning meticulously design these pathways, enabling the efficient reprocessing and strategic application of features extracted from the backbone network. Finally, to fine-tune and accurately predict the bounding box coordinates of possible objects in the input HEp-2 specimen image, the head layers are used.

The YOLOv8n model has been configured for training with specific parameters. The training process is set to run for 300 epochs without early stopping (patience=0). A batch size of 4 and an image size of 800 pixels are specified for each iteration. Employing AdamW as the optimiser and adopting a learning rate (lr) of 0.001667 alongside a momentum of 0.9, the trial-and-error method was utilised to experimentally fine-tune these hyper-parameters.

The YOLO (You Only Look Once) model, introduced in 2015, revolutionised single-shot detection. It divides the image into a k × k grid, with each grid responsible for detecting objects whose centres are within its boundaries. Multiple bounding boxes are predicted for each grid, defined by parameters like x, y (centre), width, height, and confidence. YOLO employs Intersection over Union (IoU) and non-max suppression (NMS) to select the most relevant bounding box in each grid, streamlining detection by eliminating redundant boxes. YOLOv8, released by Ultralytics in July 2023, is the latest iteration. It comprises two key components: the backbone and the head, reflecting a comprehensive design emphasising distinct functionalities.^18^

YOLOv8n utilises modified CSPDarknet53 as its backbone, YOLOv8n commences with a dual convolutional layer setup. Each convolutional layer within this structure is composed of a convolutional layer, a batch normalisation process, and the SiLU (Sigmoid Linear Unit) activation function. The YOLOv8n architecture employs a sophisticated arrangement of components for effective object detection tasks. Its Backbone, comprising a sequence of convolutional layers (P1/2 to P5/32), plays a pivotal role in extracting pertinent features from the input image. The SPPF layer, followed by convolution layers, adeptly processes features across multiple scales.

The architectural framework of YOLOv8n employs a decoupled head, distinctively segregating the tasks of classification and object detection.^16^ This design choice signifies an intentional separation of processes to enhance modularity and streamline performance. In the realm of object detection, YOLOv8n adopts the sigmoid activation function, portraying the probability of a bounding box encapsulating an object. In parallel, for classification probabilities across different classes, the SoftMax function is employed. Transitioning to the head configuration, the use of up sampling layers facilitates the restoration of higher-resolution feature maps, while concatenation with backbone features (P3, P4, P5) ensures a holistic representation. The subsequent C2f layers within the head refine these features, progressively decreasing the number of filters to distil essential information. The final segment layer amalgamates features from various scales, producing the ultimate output. This segment layer is pivotal, combining information from P3, P4, and P5, and generating the model’s predictions, including the number of classes which are 2 classes, anchor boxes (32), and dimensionality (256). The YOLOv8n’s architectural choices, such as the spatial pyramid pooling and C2f modules, contribute to its ability to detect objects with precision at different scales, making it a robust solution for object detection tasks.

#### Architecture of Detectron2

Detectron 2 stands out as a highly effective deep MASK R-CNN framework, showcasing exceptional proficiency in tasks related to object detection. It serves as a robust computer vision library, characterised by a versatile and modular architecture specifically designed for object detection and segmentation tasks. At its essence, Detectron2 employs a modular structure that segregates fundamental elements of the object detection pipeline. The backbone network is responsible for extracting hierarchical features from input images, and the Region Proposal Network identifies potential regions containing objects. Following this, ROI heads handle the processing of these regions refining box locations and classifying objects.

This network architecture consisted of ResNet with 53 layers, FPN with 14 layers, RPN with 4 layers, Stand-ardROIHeads with 4 layers, FastRCNNConvFCHead with 3 layers, FastRCNNOutputLayers with 2 layers, and MaskRCNNConvUpsampleHead with 5 layers. Detectron2 stands out as a highly proficient framework, specifically designed for tasks related to object detection.^25,26^ As a versatile computer vision library, it exhibits a modular architecture tailored for efficiency in object detection and segmentation. Introduced by Facebook AI Research in October 2019, Detectron2 serves as an upgraded version of the original Detectron framework, with a primary focus on tasks like object detection and instance segmentation.^27^ Utilising the PyTorch deep learning library, Detectron2 facilitates seamless integration with various neural network architectures, offering a conducive environment for experimentation.^27^ The framework’s four key components, including the backbone, neck, RPN region, and ROI head.

The proposed model incorporates the ResNet50 architecture as its backbone, organised into sequential stages: res2, res3, res4, and res5. Known for its depth and effective use of skip connections to address the vanishing gradient problem, this architecture starts with a Basic Stem module featuring a 7x7 convolutional layer with a stride of 2 for initial feature extraction. The Res2 stage consists of Bottleneck Blocks, strategically combining 1x1 and 3x3 convolutions to ensure efficient gradient flow and robust feature extraction. Skip connections within these blocks enhance gradient propagation during training. The use of FrozenBatch-Norm2d ensures uniform normalisation, contributing to the stability and effectiveness of feature extraction in this sophisticated model architecture.

The Feature Pyramid Network (FPN) acts as the model’s neck network, improving feature representation. Through lateral connections (fpn_lateral2 to fpn_lateral5) using 1x1 convolutions and output connections (fpn_output2 to fpn_output5) with 3x3 convolutions, the FPN adjusts channel dimensions and enhances feature maps at different scales. The LastLevelMax-Pool operation integrates the highest-level feature map, ensuring a modular and hierarchical structure. This FPN design effectively captures multi-scale features, forming a robust foundation for precise autoimmune disease cell detection.

The Region Proposal Network (RPN) analyses multi-scale features, generating around 1000 box proposals with confidence scores, representing potential bounding boxes for objects of interest. Detectron2 utilises a box head with ROI pooling to extract crucial information for class and bounding box scores from these proposals. Non-maximum suppression (NMS) is then applied, filtering proposals to a maximum of 100 boxes. The Region of Interest (ROI) Head in Detectron2 comprises two heads: the box head and the mask head. The box head, using ROI pooling, processes retained proposals for accurate class and bounding box score predictions. Simultaneously, the mask head of the ROI processes proposals with Feature Pyramid Network (FPN) feature maps, creating pixel-level segmentation masks. This two-stage process ensures a comprehensive approach to detailed pixel-level object segmentation.

The research adopts a meticulous configuration for the model in the training process. The data loader is tailored to utilise two workers, optimising the loading of training data for enhanced efficiency. Leveraging the strengths of transfer learning, Initial weights for the model are pre-trained using the COCO dataset, providing a solid foundation for feature extraction and learning. A judiciously chosen batch size of two images per iteration strikes a balance between computational efficiency and exposure to diverse training examples. The learning rate is finely calibrated at 0.000025 to navigate the delicate trade-off between rapid convergence and avoidance of overshooting optimal values. The training process is capped at a maximum of 2500 iterations, considered sufficient for the given dataset.

With a constant learning rate and a batch size per image set to 256 in the region of interest (ROI) heads, the training process is stabilised and regulated for optimal efficiency. The model is configured to recognise three classes (the background, the homogenous cells, and the mitotic cells) forming the bedrock for accurate and robust detection of objects (cells) within the specified classes. This configuration collectively establishes the framework for the model’s training process, strategically designed to achieve superior performance in pattern recognition within the target dataset.

Three sets of the dataset were created: a validation set (21%) a testing set (10%) and a training set (69%). The network was trained using the training set to change the weights. Overfitting was prevented by using the validation set. The testing set contained fresh data that the network was expected to identify. The dataset was made.

#### Loss functions of Detectron2 and YOLOv8n

Detectron2 and YOLOv8n utilise different loss functions to handle various aspects of object detection tasks. These frameworks share common components, including classification loss (loss_cls), bounding box regression loss (loss_box_reg), and mask prediction loss (loss mask). Detectron2 extends with loss_rpn_ cls and loss_rpn_loc for the Region Proposal Network, while YOLOv8n introduces Distribution Focal Loss (dfl_ loss) to address class imbalances, particularly useful in scenarios with homogenous and mitotic cells.

#### Classification loss


(1) $$\text{FL}(\text{p},\text{q})=-{\left(1-\text{p}\right)}^{\gamma }\times q\times \text{log}(p)+{\left(p\right)}^{\gamma }\times (1-q)\times \text{log}(1-p)$$

(2) $$\text{VFL}(\text{p},\text{q})={\left(1-\text{a}\right)}^{\gamma }\times {\left(\text{a}\times \text{p}\right)}^{\gamma }\times BCE(p,q)+{\left(1-\text{a}\right)}^{\gamma }\times \text{BCE}(\text{p},\text{q})$$

Detectron2 employs Focal Loss (FL) in Equation 1, while YOLOv8n uses Variant Focal Loss (VFL) in Equation 2. Both involve a combination of predicted probabilities (p), ground truth labels (q), and balancing parameters (α and γ), with BCE(p, q) representing Binary Cross-Entropy loss.

#### Bounding box regression loss

The Bounding box regression loss evaluates the disparity between the predicted and true locations of a bounding box.

(3) $$\text{DIoU}(\text{b}1,\text{b}2)=\text{IoU}(\text{b}1,\text{b}2)-p^{2}\frac{\left(\text{b}1,\text{b}2\right)}{c^{2}}$$

(4) CIoU(b1,b2)=DIoU(b1,b2)−av+v+ap×p

Equations 3 and 4 define Distance Intersection over Union (DIoU) and Complete Intersection over Union (CIoU), incorporating IoU, Euclidean distance (ρ), and additional terms for aspect ratio consistency and centre point localisation.

#### Mask prediction loss

Quantifying the accuracy of predicted binary masks, this metric assesses the model’s ability to faithfully capture and delineate intricate object boundaries.

(5) $$\text{BcE}(\text{p},\text{q})=-\sum {\text{iq}}_{\text{i}}\times \log({\text{p}}_{\text{i}})+(1-{\text{q}}_{\text{i}})\times \log(1-{\text{p}}_{\text{i}})$$

(6) $$\text{Dice}(\text{p},\text{q})=1-2\times +(1-{\text{q}}_{\text{i}})\times \frac{\left|\text{p}\cap \text{q}\right|}{\left(\left|\text{p}\right|+\left|\text{q}\right|\right)}$$

Detectron2 uses Binary Cross-Entropy (BCE) loss (Equation 5) and Dice coefficient (Equation 6) to evaluate predicted binary masks against ground truth, emphasising object boundary accuracy.

#### Region Proposal Network classification loss

The Binary Cross-Entropy Loss in the Region Proposal Network (RPN) is crucial for measuring “objectness” by evaluating the network’s proficiency in labelling anchor boxes as foreground or background within proposed regions.

(7) $$\text{CE}(\text{p},\text{q})=-\sum {\text{iq}}_{\text{i}}\times \log({\text{p}}_{\text{i}})$$

Detectron2 employs Binary Cross-Entropy Loss (CE) to measure “objectness” in the Region Proposal Network as shown in Equation 7.

#### Region Proposal Network localisation loss

The localisation loss in the Region Proposal Network (RPN) assesses the accuracy of predicted region local-isations, guiding the network for precise spatial coordinate determination during object detection.

(8) $$\text{Smooth}L1(\text{x})=\left|\text{x}\right|\text{i}f\left|\text{x}\right|<1.0.5\times {\text{x}}^{2}+0.5$$

Equation 8, Smooth L1 loss, is a versatile function for localisation tasks. It computes the element-wise difference between predicted and ground truth bounding box coordinates.

#### The Distribution Focal Loss

The Distribution Focal Loss (DFL) is crucial in training object detection models, especially in scenarios with imbalanced class distributions. DFL is associated with YOLOv8n only.

(9) $$\text{DFL}=-{\left(1-\text{pt}\right)}^{\gamma }\times \log(\text{pt})\times P{\text{enalty(predicted}}_{\text{distribution}},{\text{ground}}_{{\text{truth}}_{\text{distribution}}})$$

Exclusively in YOLOv8n, DFL (Equation 9) incorporates focal terms, fine-tuning weights based on predicted probabilities, and a penalty function to assess dissimilarity between predicted and ground truth distributions.

#### Total loss

It is the summation of all the loss functions present in the detectron2 model.

(10) $$\begin{array}{l} \text{total}\_\text{loss}=\text{wcls}\times \text{cls}\_\text{loss}+\text{wbox}\_\text{reg}\times \\ \text{loss}\_\text{box}\_\text{reg}+\text{wrpn}\_\text{cls}\times \text{loss}\_\text{rpn}\_\text{cls}+\\ \text{wrpn}\_\text{loc}\times \text{loss}\_\text{rpn}\_\text{loc}+\text{wmask}\times \text{loss}\_\text{mask} \end{array}$$

Here, wcls, wbox_reg, wrpn_cls, wrpn_loc, and wmask represent respective weight factors assigned to each loss component.

## EXPERIMENTS AND RESULT

The performance of deep learning algorithms in detecting Human Epithelial-type 2 (HEp2) especially homogenous cells and mitotic cells from annotated data was assessed using metrics like Mean Average Precision (mAP), Precision, Recall, Average Precision (AP), and Average Recall (AR). These metrics gauge the accuracy and effectiveness of models like YOLOv8 and Detectron2. mAP averages the precision-recall trade-off for detecting different cell categories, while Precision measures the accuracy of predicted detections. Recall evaluates the model’s ability to capture all actual instances. AP quantifies precision-recall trade-offs across various thresholds, and AR computes the average recall across different recall values. These metrics provide valuable insights into the performance of object detection algorithms, aiding in assessing their suitability for real-world applications.

The equations for these metrics are as follows:

Mean average precision (mAP)

(11) $$mAP=\frac{1}{N}\sum_{i=1}^{N}AP_{i}$$

Precision

(12) $$\mathrm{Precision}=\frac{TP}{TP+FP}$$

Recall

(13) $$\mathrm{Recall}=\frac{TP}{TP+FN}$$

Average Precision (AP)

(14) $$AP=\sum_{k=0}^{n-1}\left[\mathrm{Recall}(k)-\mathrm{Recall}(k+1)\right]*\mathrm{Precision}(k)]$$

Average Recall (AR)

(15) AR=n1∑k=0n−1Recall(k)

These studies aimed to develop automated systems for identifying cells associated with autoimmune diseases, using advanced computer algorithms to recognise specific cell patterns in microscopic images. Both methods focused on detecting uniform and mitotic cell patterns in HEp-2 cell images, with one study proposing a deep learning framework and the other implementing meticulous manual annotation techniques to enhance efficiency in annotating these cells. The results revealed that the YOLOv8n model outperformed the Detectron2 model in accurately identifying these cells, demonstrating superior accuracy in predicting cell shapes and creating detailed cell outlines. While these findings hold promise for improving autoimmune disease cell detection, caution is warranted in practical application until further research confirms the reliability of these models across different scenarios. Overall, these studies offer new avenues for enhancing the detection of autoimmune disease cells, highlighting the importance of ongoing refinement and validation efforts to ensure their effectiveness in real-world applications.

### Hyperparameters Configuration of Detectron2 and YOLOv8n during training

The configuration parameters for training the Detectron2 model exhibit a meticulous optimisation strategy. The effective batch size of two images per iteration strikes a balance, offering computational efficiency while ensuring exposure to diverse training examples. The learning rate, set at 0.000025, reflects a careful calibration to navigate the delicate trade-off between achieving rapid convergence and avoiding overshooting optimal values. The training process is capped at a maximum of 2500 iterations, a number deemed sufficient for the given dataset. Utilising Stochastic Gradient Descent (SGD) with momentum, the learning rate remains constant throughout training, and no decay steps are implemented. Within the region of interest (ROI) heads, a batch size per image of 256 is chosen to optimise efficiency. The model, designed to recognise three classes—background, homogenous cells, and mitotic cells—provides a robust foundation for accurate object detection within the specified classes, contributing to the overall efficacy and precision of the training process with a total number of 43928802 of parameters being trained.

The YOLOv8n model has undergone meticulous configuration for optimal training, with a prescribed duration of 300 epochs and a strategic exclusion of early stopping (patience=0). Each training iteration is structured with a batch size of 4 and a standardised image size of 800 pixels. The utilisation of the AdamW optimiser, accompanied by a learning rate (lr) of 0.001667 and a momentum setting of 0.9, was determined through a systematic trial-and-error approach to refine these critical hyperparameters. Notably, the model comprises a substantial 43,928,802 parameters, which were subjected to the training process to enhance the detection capabilities for autoimmune disease cells. This rigorous configuration is aligned with the overarching objective of leveraging YOLOv8n and Detectron2 for precise and efficient detection of autoimmune diseases. Three sets of the dataset were created: a validation set (21%) a testing set (10%) and a training set (69%). Both model’s networks were trained using the training set to change the weights. Overfitting was prevented by using the validation set. The testing set contained fresh data that the network was expected to identify. The dataset was made.

## EXPERIMENT- 1

In the experiment, the Detectron2 model was utilised for the detection of homogenous and mitotic cells. The model was trained on a modest dataset comprising 72 images, utilising an 82-layer network architecture. The model, with a total of 43,928,802 parameters, underwent rigorous training to effectively learn the complex patterns associated with autoimmune cells. The model’s performance was assessed on a validation set after training in which the validation set consisted of 22 images and was subsequently tested on a separate set of 10 images individually.

The Detectron2 model’s performance was assessed using COCO metrics.

**Figure 5.**
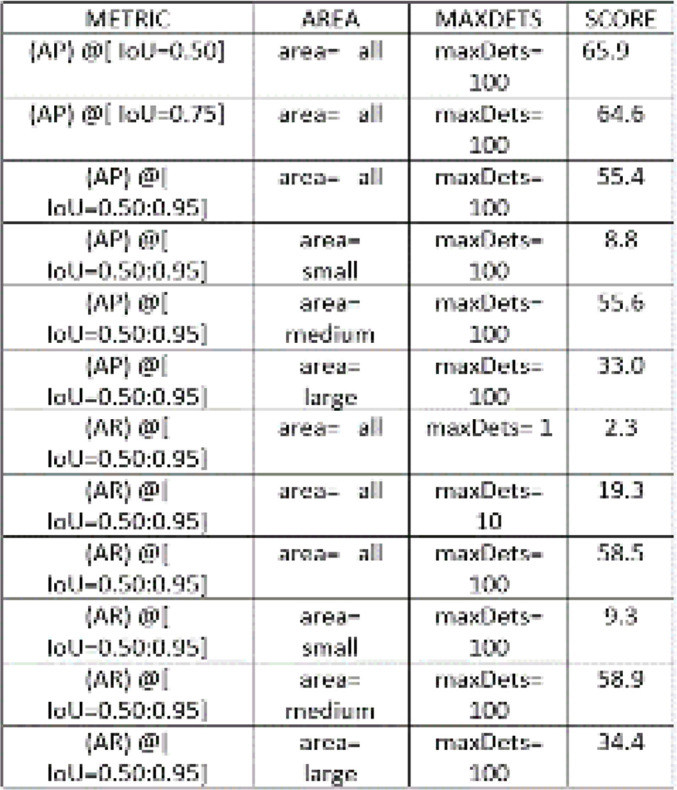
AP and AR for bounding boxes at different Intersections over Union thresholds and cell sizes.

**Figure 6 (left).**
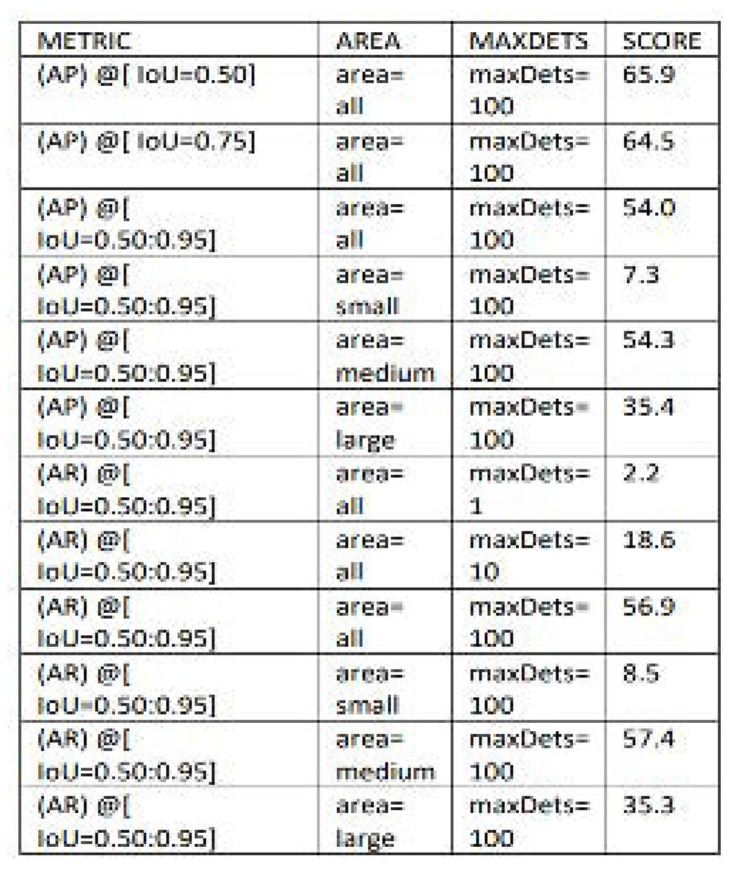
AP and AR for segmentation mask (segm) at different Intersections over Union (IoU) thresholds and cell sizes.

**Figure 5** provides the assessment encompassed the computation of essential metrics pertaining to bounding boxes (bbox) across a spectrum of Intersection over Union (IoU) thresholds and distinct object sizes. The overall Average Precision (AP) have obtained for bounding boxes is 55.370% and overall Average Recall (AR) is 58.53% and the term “maxDets” in **Figure 6** pertains to the maximum number of detections taken into account when evaluating the model’s performance. **Table 2** encapsulates the comprehensive results, presenting the categorically defined Average Precision (AP) scores for both bounding boxes (bbox) and segmentation masks (segm) across class A and class B. Additionally, the mean Average Precision (mAP) scores for both classes are included in the Figure.

**Table 2.** Overall performance for both class A and class B in Experiment 1.

**METRIC**	**AP-A**	**AP-B**	**mAP**
bbox	62.01	48.73	55.36
segm	61.16	46.86	54.00

Here class A represents the homogenous cell and class B represents the mitotic cells that are present in the input images and overall mAP was 54% for segmentation mask and 55% for bounding box.

**Figure 7 (below).**
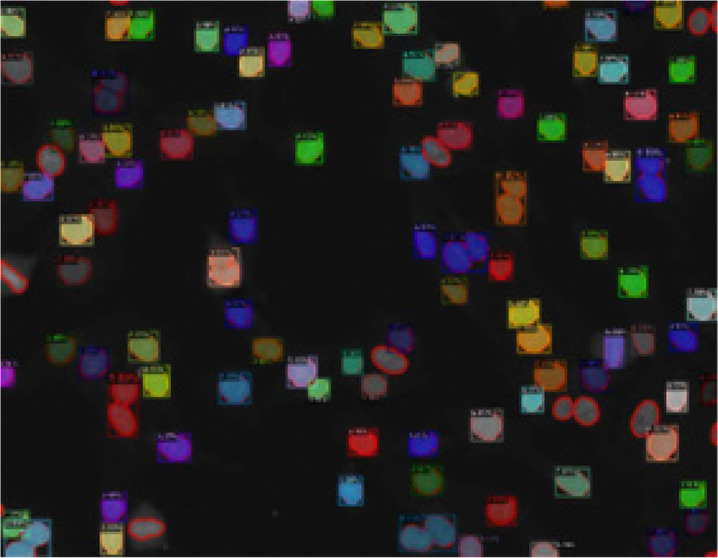
Detection Output from the Detectron2 Model.

Detectron2 was successfully able to detect 91 class A out of 104, but it was successfully able to detect all the class B (which is 9 out of 9) in **Figure 7**.

**Table 1.** Hyperparameters configuration of Detectron2 and YOLOv8n during training.

**Component**	**Value**
Learning Rate	0.001667
Epochs	300
Batch Size	4
Momentum	0.9
PCA Components	NA
ReliefF Top Features	NA
Number of Model Parameters	43,928,802
Loss Function	Binary cross-entropy
Optimiser	AdamW optimiser
Deep Learning Architecture	YOLOv8n (261 layers, 3,409,968 parameters, with enabled gradient parameters) Detectron2 (Pre-trained models on PyTorch)
Computational Efficiency	YOLOv8n: 12.8 GFLOPs
Training Procedures	- Dataset Preparation: Training, validation, and testing sets - Model Initialisation: Pre-trained weights - Fine-Tuning Optimisation: SGD, learning rate scheduling

## EXPERIMENT- 2

Experiment 2 aimed to illustrate the effectiveness of the suggested approach YOLOv8n algorithm in the detection of homogenous cells and mitotic cells on the complete dataset. The dataset comprised 104 HEp-2 cell samples. For training purposes, 69% (72) of the dataset was utilised, while the remaining 21% (22) served for validation and testing. And 10% (10) for each was used. YOLOv8n model comprised 261 layers with a total of 3,409,968 parameters and corresponding gradients. With a computational intensity of 12.8 GFLOPs (Giga Floating-point Operations Per Second).

**Figure 8.**
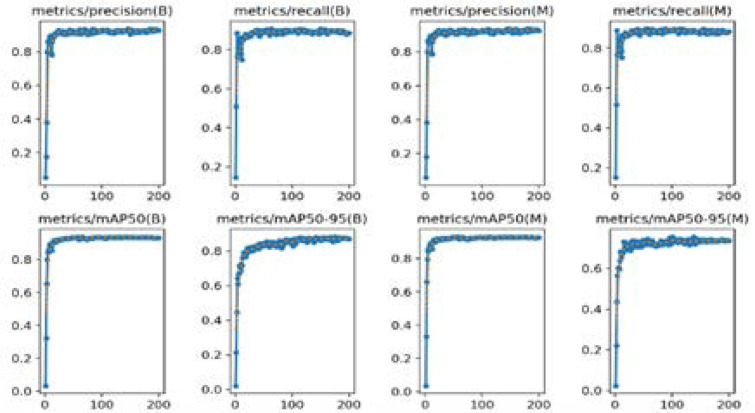
The validation performance concerning map, precision, and recall.

In **Figure 8**, the graph plotted the relationship between precision, recall, mAP50, and mAP50-95 metrics against bounding boxes and masks over 200 epochs. **Table 3** presents an overview of evaluation metrics for bounding boxes and masks on different classes (all, A, B) within the dataset. For each class, **Table 3** provides information on the precision (P) and recall (R) for bounding boxes and masks. The metrics include mAP50 and mAP50-95, which measure the average precision at different IoU thresholds. The results indicate the model’s performance in accurately identifying instances and delineating variations across different classes.

**Table 3.** Overall performance for both class A and class B in Experiment 1.

**CATEGORY**	**BOUNDING BOXES**				**MASKS**								
**METRICS**	**PRECISION**	**RECALL**	**mAP50**	**mAP50-95**	**PRECISION**	**RECALL**	**mAP50**	**mAP50-95**
All	92.8	88.7	94	87.2	92.4	88.3	93.4	75.7
A	94.6	93.3	97.6	90.1	94.1	92.8	96.6	75.6
B	91	84	90.5	84.3	90.8	83.8	90.2	75.8

YOLOv8n demonstrated a commendable overall mAP score of 94% for bounding box and 93% for segmentation mask, underscoring its practical utility for addressing real-world problems.

**Figure 9.**
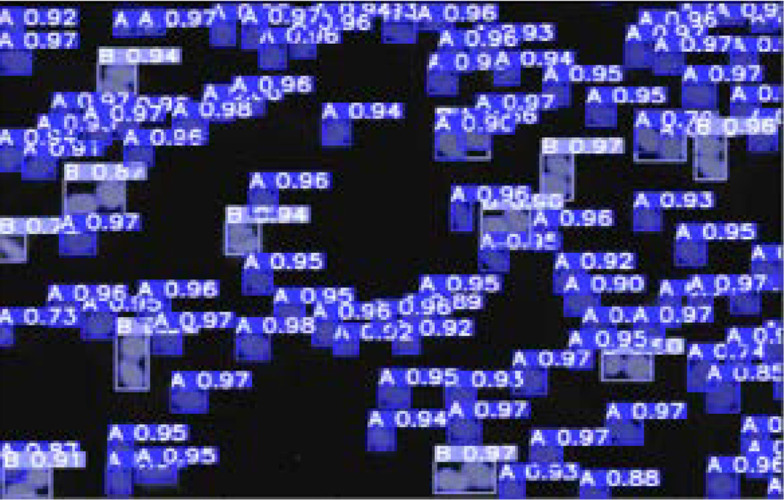
Detection Output from the YOLOv8n Model.

YOLOv8n was successfully able to detect 98 class A out of 104, but it was successfully able to detect all the class B (which is 9 out of 9) in **Figure 9**.

## DISCUSSION

This study aimed to develop a dependable automated procedure for identifying cells linked to autoimmune diseases, with a specific focus on recognising uniform and mitotic cell patterns in HEp-2 cell images. The automatic deep learning framework presented in this paper can identify homogenous cells and mitotic cells directly from the microscopic images of HEp-2 specimens. To overcome the scarcity of labeled datasets, we’ve implemented a rigorous manual annotation method to improve the efficiency of annotating uniform and mitotic cells in images. This enhancement aims to boost the detection performance by incorporating supervised deep learning models.^19^ Experimental results revealed that, in this particular task, the YOLO-based framework outperformed its Detectron2 counterpart.

Previous machine learning methods for autoimmune disease diagnosis might have struggled with feature extraction and robustness. The deep learning techniques employed in the paper, particularly the YOLOv8 model, offer a more effective approach to feature extraction and improved model robustness. This ensures a higher accuracy rate in identifying relevant patterns associated with autoimmune diseases in human epithelial cells.

Some of the previous methods used in this field were: Deep Convolutional Neural Networks (DCNN) which can be prone to overfitting when trained using limited training data whereas YOLOv8 employs data augmentation techniques during training to address overfitting concerns.^2^

Segmentation Guided HEp-2 Cell Classification Method via GANs where its effectiveness may heavily rely on segmentation masks generated by GAN. The training process involved a considerable number of parameters for both the Generative Adversarial Network (GAN) and YOLOv8. However, it’s essential to note that YOLOv8 does not inherently incorporate GANs for object detection. It focuses on the one-stage object detection task using the anchor-based approach. This simplifies the training process, making it more stable and straightforward compared to GAN-based methods.^2^ ResNet-50 can face challenges such as the degradation problem whereas YOLOv8 uses CSPDarknet53 as its backbone, which is an improvement over the traditional ResNet-based backbones. This architecture helps mitigate issues related to vanishing gradients in very deep networks and contributes to the stability and effectiveness of training.^17^ The use of deep architectures like Deeplabv3 plus with Exception can be computationally intensive, potentially limiting real-time applications on less powerful hardware.^5^

YOLOv8 directly addresses object detection tasks and does not explicitly rely on segmentation accuracy. It focuses on predicting bounding boxes and class probabilities for objects in the entire image, rather than segmenting individual objects. Moreover, YOLOv8 is designed to strike a balance between accuracy and computational efficiency. By utilising optimised architectures and techniques, YOLOv8 aims to deliver good performance on various hardware platforms, including edge devices. While the mentioned methods focus on image classification and segmentation in the context of HEp-2 cell images, YOLOv8 (You Only Look Once version 8) is a state-of-the-art object detection model. YOLO models are designed to detect and locate objects within an image, providing bounding box coordinates and class probabilities for each object.^28,29^

The performance evaluation indicates that the YOLOv8n model outperformed the Detectron2 model in both tasks of predicting bounding boxes and generating segmentation masks. Specifically, the accuracy improvement was notable, with a 70.12% enhancement in bounding box prediction and a substantial 72.78% improvement in segmentation mask creation. These results underscore the efficacy of the YOLOv8n model in comparison to the Detectron2 model across these two critical tasks. The incorporation of the YOLOv8n model holds the potential for enhancing the precision of autoimmune disease cell detection in future research and applications. It is essential to note, however, that practical application should proceed with caution until additional data is available and further research is conducted to establish the model’s robustness and reliability in varied scenarios. The deployment of the YOLOv8n model in practical situations should be contingent upon the accumulation of more comprehensive datasets and thorough investigation to ensure its efficacy across diverse conditions and applications.

### Comparison of the state-of-art method

Detectron2 and YOLOv8n were selected for their state-of-the-art status, adaptive architectures, real-time efficiency, and superior performance in medical imaging. These models have demonstrated exceptional capabilities in accurately segmenting Hep2 cells in auto-immune disease detection, underscoring their importance in advancing research in this domain, as shown in **Table 4**. Their advanced segmentation abilities facilitate precise delineation of intricate cell structures and accommodate staining variations in medical images effectively.

**Table 4.** Detection Output from the Detectron2 Model.

**Metrics**	**YOLO v8**		**Dectecron2**	
	**mAP**		**mAP**	
	**Bounding Box**	**Seg Mask**	**Bounding Box**	**Seg Mask**
A	97.6	96.6	62.01	61.16
B	90.5	90.2	48.72	46.86
All	94	93.4	55.36	54.00

Compared to alternatives such as YOLOv5, UNet, and Mask R-CNN, Detectron2 and YOLOv8 stand out due to their advanced backbone architectures and robust mask prediction capabilities. While YOLOv5 shares some efficiency aspects with YOLOv8, the latter typically outperforms in instance segmentation tasks. UNet, although popular, may struggle to capture multi-scale features efficiently, potentially resulting in suboptimal results in complex medical images. Similarly, while accurate, Mask R-CNN may require more computational resources compared to YOLOv8 and Detectron2, rendering them less suitable for resource-constrained medical imaging environments.

In the realm of autoimmune disease detection within human epithelial cells, the evaluation based on Mean Average Precision (mAP) unequivocally positions YOLOv8 as a superior method when compared to Detectron 2. YOLOv8, renowned for its real-time object detection capabilities, not only demonstrates competitive mAP scores but also excels in the crucial balance between speed and accuracy. The integration of YOLOv8, with its single-pass architecture and efficient feature extraction, emerges as a transformative approach. The fusion of deep learning techniques within YOLOv8 not only elevates its precision in localising autoimmune disease indicators but also accelerates the detection process. This research underscores YOLOv8 as the optimal choice for autoimmune disease detection, emphasising that the amalgamation of cutting-edge deep learning methodologies has propelled it to the forefront, offering rapid and accurate identification of disease-related features in scanned images of human epithelial cells.

The comparison between YOLOv8n and Detectron2 in the Hep2 cell instance segmentation project for auto-immune disease detection reveals YOLOv8n’s superior performance, attributed to its advanced features. YOLOv8n employs a distributed focal loss mechanism, adjusting class weights dynamically during training to tackle common class imbalance challenges in medical imaging tasks. This adaptive approach enhances its accuracy in detecting rare yet clinically significant patterns indicative of autoimmune diseases.

Moreover, YOLOv8n integrates state-of-the-art data augmentation techniques such as mix-up and Mosaic. Mix-up blends multiple images to diversify the training dataset, fostering improved generalisation while mitigating overfitting risks. Mosaic augmentation further enhances adaptability by exposing the model to diverse scenarios with varying object compositions and spatial relationships. This combined use of mix-up and Mosaic techniques bolsters YOLOv8n’s robustness in instance segmentation, particularly in the challenging context of autoimmune disease Hep2 cell images. Additionally, YOLOv8n’s finely tuned architecture for medical image instance segmentation excels in capturing intricate cell structures and staining patterns, contributing to its superior performance. Its unified approach to object detection and instance segmentation enhances efficacy in autoimmune disease detection scenarios compared to the potentially less synergistic modular approach of Detectron2.

Expanding upon the discussion, acknowledge the need to explore the broader implications of the findings for autoimmune disease diagnostics. The study primarily utilised homogeneous cells, and recognise the importance of extending the research to encompass a more diverse cell population. In future work, the inclusion of various cell types would offer a more comprehensive understanding of the model’s applicability across different autoimmune diseases. Additionally, the research has meaningful implications beyond the scope of this study. The adaptability of the model positions it as a valuable tool in the medical field, potentially streamlining the diagnostic process and contributing to advancements in autoimmune disease research.

It is crucial to address the limitations of the study. One notable limitation is the focus on homogeneous cells, excluding consideration for other cell types that may exhibit different staining patterns. Future studies should aim to encompass a more diverse cell population to enhance the model’s applicability across a broader spectrum of autoimmune diseases. In terms of generalisability, the model demonstrates robustness and impartiality. Although initially trained on the ICPR 2016 dataset, the model underwent validation using the dataset, The absence of bias in the model after this validation process underscores its generalisability across different datasets and imaging modalities, instilling confidence in its potential application in various clinical settings.

The advantages of automating the diagnosis include increased efficiency, accuracy, and consistency in disease detection. By leveraging advanced deep learning frameworks like YOLOv8n and Detectron2, automated diagnostic systems can analyse medical images with precision, minimising human error and variability in interpretation. This consistency is paramount for reliable and reproducible diagnoses, which is essential in autoimmune disease diagnostics where subtle patterns in cell morphology may indicate disease presence or progression.

Furthermore, automation enables early detection of abnormalities, leading to timely interventions and improved patient outcomes. Automated diagnostic systems are also scalable, allowing healthcare facilities to handle larger volumes of medical data efficiently, especially in regions with limited access to healthcare centres. Although initial implementation costs may be involved, automated diagnostic systems ultimately offer cost savings by reducing the need for manual labour and streamlining workflow processes. Additionally, automated systems provide valuable decision support to healthcare professionals by presenting them with objective data and insights derived from medical images. This support enhances clinical decision-making, leading to more informed treatment plans and personalised patient care. Integration with electronic health record systems further enhances care coordination and facilitates comprehensive patient management.^18^

In the coming time, the project could be developed by identifying relevant datasets, or collaborating with institutions like Ramaiah Medical College which has insights on different autoimmune diseases or cell types to develop more robust model, along with engagement with domain experts to understand specific requirements for each disease or cell type. There would be a need to develop transfer learning strategies to adapt pre-trained models to new disease contexts, and fine-tuning models on disease-specific datasets while preserving the knowledge gained from the original training on autoimmune diseases. Data preprocessing pipelines could be developed which will handle different data formats and structures, and techniques for data augmentation implemented to increase dataset diversity. Finally, compatibility with the chosen deep learning frameworks (e.g., PyTorch for YOLOv8, Detectron2) must be ensured.

In the future, experimentation with model architectures, hyperparameter tuning, and novel training strategies can be implemented. Leveraging more extensive and diverse datasets for training may also be considered. There is a scope to explore the incorporation of additional data modalities, such as genetic information, patient history, or other diagnostic tests, to enhance the overall diagnostic capabilities. This can be achieved by developing fusion models that can effectively integrate information from multiple sources and ensure interoperability with existing medical data systems.

## CONCLUSION

This research paper introduces an innovative automated approach to Human Epithelial -type 2 cell segmentation and classification. A comprehensive methodology integrating segmentation-guided classification, deep learning frameworks, and advanced image processing techniques was proposed.

The preprocessing pipeline ensures the standardisation and normalisation of a diverse dataset of scanned HEp-2 cell grayscale images. Through thoughtful annotations and dataset division, the class imbalances were addressed, enabling effective training of the deep learning models. Leveraging YOLOv8n and Detectron2, introducing a resilient process for diagnosing autoimmune diseases, with a focus on identifying uniform and dividing cells.

Proposed deep learning models undergo meticulous training, hyperparameter fine-tuning, and evaluation, showcasing their efficiency and accuracy in detecting Homogenous rim HEp-2 cells and mitotic cells. The incorporation of a cell counting step provides a quantitative understanding of disease manifestation, contributing to the depth of the proposed workflow.

In Experiment 1, utilising the Detectron2 model achieved an overall mAP score of 55.36% for bounding boxes and 54.00% for segmentation masks.

In Experiment 2, employing the YOLOv8n model attained an impressive overall mAP score of 94% for the bounding box and 93% for the segmentation mask, showcasing its efficacy in detecting HEp-2 cells.

The research contributes to the ongoing transformation in autoimmune disease diagnostics, offering a reliable and automated approach for identifying HEp-2 cell patterns. The suggested framework exhibits its proficiency and dependability, setting the stage for further advancements in the field.

## CONFLICT OF INTEREST

The authors declare no conflict of interest.
